# Effects of a basketball intervention on female college students' positive body image: chain mediated role of the experience of embodiment and self-compassion

**DOI:** 10.3389/fpsyg.2025.1593420

**Published:** 2025-09-04

**Authors:** Huachen Zhu, Yuhong Wen

**Affiliations:** ^1^School of Recreational Sports and Tourism, Beijing Sport University, Beijing, China; ^2^Key Laboratory of Sport Training of General Administration of Sport of China, Beijing Sport University, Beijing, China

**Keywords:** exercise intervention, basketball, positive body image, the experience of embodiment, self-compassion

## Abstract

This study investigated how basketball intervention affects positive body image in female college students, examining the mediating roles of the experience of embodiment and self-compassion. A randomized controlled trial design was adopted, in which forty two participants were randomly assigned to either a 10-week basketball intervention program (conducted twice weekly, each lasting 90 m) or a standard physical education curriculum that included aerobics, Tai Chi, and physical fitness exercises. Data were collected using validated scales measuring the experience of embodiment, self-compassion, body appreciation, and functionality appreciation. The data analysis included repeated measures ANOVA, mediation effect testing, and machine learning models. The basketball intervention produced significant main effects of time, group, and their interaction on the experience of embodiment, self-compassion, and positive body image among female college students. The chain mediation analysis revealed that both the experience of embodiment and self-compassion mediated the relationship between the basketball intervention and positive body image. Specifically, the mediation effects were as follows: the experience of embodiment accounted for 2.73%, self-compassion for 33.61%, and the chain mediation for 11.98%. Participation in both the basketball group and the comparison group was found to enhance the experience of embodiment, self-compassion, and positive body image. However, the basketball intervention produced significantly more pronounced effects compared to the mixed-activity standard physical education program. It directly improved positive body image and also exerted indirect effects through the independent mediating role of self-compassion as well as the sequential mediation of the experience of embodiment and self-compassion.

## 1 Introduction

The body, serving as a medium, interacts with the surrounding environment and thus influences psychological states. In this context, body image, which encompasses an individual's perception and experience of their own body, involves not only the perception of physical appearance but also experiences related to physical functionality and biological integrity (Cash, 2012). Body image can be categorized into positive and negative dimensions. Physical exercise, recognized as a proactive health behavior, has been empirically demonstrated to mitigate depression (Kvam et al., 2016), alleviate anxiety (Singh et al., 2023), and reduce feelings of inferiority (Liu, 2022), among other negative psychological states. It also helps enhance self-esteem (Ouyang et al., 2020), boost confidence (Soulliard et al., 2019), and increase subjective well-being (Buecker et al., 2021). Building on this foundation, previous studies have established and explored the relationship between physical exercise and positive body image. The motivation, form, environment, and frequency of sports activities are all significantly correlated with positive body image. Specifically, health-focused exercise motivation (Tylka and Homan, 2015), engagement in natural exercise environments (Baceviciene et al., 2021), and maintaining a high exercise frequency (Levenig et al., 2020) all show significant positive associations with positive body image.

Although existing studies have confirmed that physical exercise can promote positive body image, they have primarily focused on mindfulness-based yoga programs (Halliwell et al., 2019; Kramer and Cuccolo, 2020) and aesthetic dance programs (Suskin and Al-Yagon, 2020). However, research has shown that the structural social support inherent in team sports enables individuals to alleviate the pressure of singular aesthetic standards through peer cohesion and collective goal pursuit, thereby directly fostering positive body image (Jankauskiene et al., 2024). Basketball, as a group sport that combines aerobic and anaerobic elements, not only reduces negative emotions but also enhances embodiment through shared agency and mutual reinforcement (Brunet et al., 2010). Empirical studies have demonstrated that university women participating in team sports show significantly higher levels of body appreciation due to reduced self-objectification and strengthened group identity (Sabiston et al., 2018). An 8-week basketball intervention for overweight female middle school students has been shown to significantly enhance self-awareness, particularly in the dimensions of physical appearance and attributes (Li, 2019). These findings suggest that basketball programs may contribute to the enhancement of positive body image among female college students. Additionally, gender differences in positive body image have been widely confirmed, with males reporting significantly higher average levels of positive body image than females (Linardon et al., 2022, 2023). In school physical education, girls in single-gender teaching environments are also able to sustain moderate-to-vigorous physical activity for longer periods and demonstrate more positive exercise performance (Wallace et al., 2020).

It is worth noting that in the process of physical exercise promoting positive body image, both the experience of embodiment (Tiggemann et al., 2011) and self-compassion (Yang, 2014) have been found to play mediating roles. In psychological research, the experience of embodiment refers to the life experiences individuals acquire through their bodies interacting with the environment (Piran, 2016). This concept should be clearly distinguished from embodiment itself. While embodiment refers to the fundamental condition of human existence as inherently bodily (Gallagher, 2005), the experience of embodiment denotes its subjective, contextualized manifestation through sensory-motor engagement (Piran, 2017). As individuals perceive the external world, they acquire experience, knowledge, and emotion through bodily interaction with their surroundings. Grounded in the Developmental Theory of Embodiment (DTE) (Piran, 2016), enjoyable physical activities not only strengthen the mind-body connection but also foster a positive experience of embodiment. This sense of capability can stimulate individuals' awareness and attention to their own bodies. More specifically, the experience of embodiment developed through physical activities may promote embodied presence, thereby reinforcing the mind-body connection, reducing self-objectification tendencies, and ultimately contributing to an improved body image (Theberge, 2003). Empirical evidence shows that athletes often demonstrate higher levels of positive body image than non-athletes, partly due to the unique experience of embodiment in sports, which fosters empowerment, mitigates objectifying experiences, and reduces the reinforcement of self-objectification processes (Tiggemann et al., 2011).

Self-compassion involves being open to one's own suffering, rather than avoiding or disconnecting from it, and generating the desire to alleviate suffering and heal oneself with kindness (Neff, 2003a). Research suggests that self-compassion, as an emotional resource, helps individuals overcome challenges and obstacles in sports (Zhang et al., 2023). For instance, studies have found a significant positive correlation between self-compassion and physical activity among postpartum women, suggesting that self-compassion helps facilitate their adaptation to motherhood and maintain their exercise routines (Kullman et al., 2021). Meanwhile, Wong et al., through their application of the revised Exercise and Self-Esteem Model with Self-Compassion (EXSEM-SC), demonstrated that physical exercise exerts both direct and indirect effects on self-compassion (Wong et al., 2023) and serves as an effective intervention to enhance self-compassion and mental wellbeing (Wong et al., 2021a). The experience of embodiment and self-compassion may thus be two key psychological dimensions in understanding the internal mechanisms through which physical exercise improves positive body image. While the experience of embodiment helps explain the cognitive transformation of body image in sports, self-compassion plays a role in alleviating negative emotions during exercise and enhancing motivation.

In summary, this study proposes three hypotheses. First, basketball intervention enhances female college students' experience of embodiment, self-compassion, and positive body image (H1). Second, basketball intervention indirectly influences positive body image through the separate mediating roles of the experience of embodiment and self-compassion (H2). Third, the experience of embodiment and self-compassion play a chain-mediating role in the pathway through which basketball intervention improves positive body image (H3).

## 2 Material and methods

### 2.1 Participants

Using G^*^Power 3.1.9.7 software, the required sample size was calculated to achieve 80% statistical power with a significance level (Alpha) of 0.05, an effect size of 0.25, and a non-sphericity correlation (Corr among rep Measures) of 0.5. The minimum sample size required was calculated to be thirty four participants. To account for potential attrition, the minimum effective sample size for this study was set at forty participants. Anyang Normal University and Anyang Institute of Technology were specifically selected based on their regional representativeness within Henan Province and the alignment of their physical education curricula with the National Physical Education Curriculum Guidelines for General Universities issued by the Ministry of Education. These institutions also offered feasible access for recruitment and implementation under consistent instructional and administrative conditions, which facilitated the comparability of intervention outcomes. Inclusion criteria were: (1) full-time undergraduate students; (2) biologically female; (3) aged 18–25; (4) a score ≤ 3.7 on the positive body image scale; (5) no physical activity limitations; (6) no eating disorders; (7) no clinically defined cognitive or psychological disorders; (8) no intellectual disabilities or reading comprehension difficulties; (9) voluntary participation with informed consent. Exclusion criteria included: (1) history of major illnesses or surgeries; (2) specialized sports experience of 1 year or more; (3) students majoring in sports-related fields. A total of forty two eligible participants were recruited. They were first ordered chronologically by enrollment time. A set of forty two random numbers was generated using a random number generator, then assigned to participants in sequence. The participants were sorted in ascending order based on their assigned random numbers. The top twenty one were allocated to the experimental group and the bottom twenty one to the control group. All participants provided written informed consent. The study was conducted in accordance with the Helsinki Declaration and received ethical approval from the Ethics Committee of the Sports Science Experiment at Beijing Sport University (No.: 2025014H).

### 2.2 Intervention

This study adopted a 2 (basketball group, control group) × 2 (pre-test, post-test) mixed experimental design, with participants randomly assigned to either the experimental group or the control group. The experimental group engaged in a basketball intervention program twice a week for 90 m per session over a 10-week period, in addition to their regular physical education classes. The comparison group attended only their regular physical education classes, which included activities such as aerobics, Tai Chi, and physical fitness exercises. The basketball intervention program was designated as the independent variable, while the experience of embodiment and self-compassion were considered mediating variables, and positive body image was identified as the dependent variable.

The design of the basketball intervention units and the psychological assessment objectives for the experimental group were informed by the *Self-Compassion Training Program* (Neff and Germer, 2013) and the *Handbook of Positive Body Image and Embodiment: Constructs, Protective Factors, and Interventions* (Piran, 2019), which outlines a school-based curriculum to foster positive body image. The intervention comprised 10 thematic units: team building, self-kindness, emotional regulation, self-appreciation, self-acceptance, developing self-esteem and confidence, embracing imperfection, facing interpersonal challenges, mind-body coordination, and embracing the future, with each unit containing 2 sessions. The basketball intervention content was developed based on authoritative instructional materials, including *Ball Games: Basketball* (Wang, 2015), *Strength training for basketball* (Gillett and Burgos, 2020), and *Basketball Basic Skills Tutorial* (Hu and Li, 2021). These sources were synthesized to systematically integrate theoretical knowledge and key guidance on fundamental basketball skills and tactics. The program covered core knowledge areas such as basic basketball principles, stationary dribbling, physical challenges, and was structured into four modules: basic knowledge, fundamental skills, tactical application, and exhibition or competition, as detailed in Table 1. Building upon prior research on sports-based interventions for college students' physical and mental wellbeing, the program was specifically tailored to the daily routines, physical and psychological characteristics, and practical needs of Chinese female college students, with the aim of promoting a more positive body image.

**Table 1:** Distribution of sessions for the basketball module.

**Modules**	**Basic knowledge**	**Fundamental skills**	**Tactical application**	**Exhibition or competition**
Sessions	2	11	3	4

### 2.3 Measures

A paper-and-pencil psychological assessment was conducted on-site, with standardized instructions provided to ensure that participants completed questionnaires based on their actual experiences. All completed questionnaires were collected immediately after the session. Each participant simultaneously completed 5 distinct scales: the Physical Activity Rating Scale, the Experience of Embodiment Scale, the Self-Compassion Scale, the Body Appreciation Scale, and the Functionality Appreciation Scale. Notably, the Physical Activity Rating Scale was used as the primary screening tool to identify participants with typical levels of physical activity.

#### 2.3.1 Physical Activity Rating Scale-3

The study utilized the Physical Activity Rating Scale-3 (PARS-3), revised by (Liang 1994), which was adapted and translated from an original scale developed by Japanese scholar Hashimoto Kimio. This instrument comprises three items that evaluate participants' physical activity levels in terms of intensity, duration, and frequency, based on a 5-point Likert scale. Physical activity levels were calculated using the formula: Physical Activity Participation = Intensity × (Duration-1) × Frequency (with scores ranging from a minimum of 0 to a maximum of 100). Based on the scores, activity levels were categorized as low ( ≤ 19 points), moderate (20–42 points), and high (≥43 points). The measurement outcomes demonstrated that the K-S non-parametric test indicated statistical significance (*p* < 0.001, *df* = 3), and the overall Cronbach's α coefficient was 0.683.

#### 2.3.2 Experience of Embodiment Scale

The study utilized the Chinese version of the Experience of Embodiment Scale (EES) adapted by Tian et al. (2024) from the original scale developed by Piran et al. (2020). The scale consists of 31 items, evaluating six dimensions: positive body connection and comfort, body unencumbered adjustment, agency and functionality, experience and expression of sexual desire, attuned self–care, and resisting objectification. A Likert 5-point scale was used, ranging from “strongly disagree” to “strongly agree.” Items 3, 4, 7, 10, 12, 13, 15, 16, 18, 19, 21, 23, 27, 28, 30, and 31 were reverse-scored, with the total score reflecting the level of the experience of embodiment. The measurement outcomes demonstrated that the K-S non-parametric test indicated a significant level (*p* < 0.001, *df* = 465), and the overall Cronbach's α coefficient was 0.694.

#### 2.3.3 Self-Compassion Scale

The study utilized the Adolescent Self-Compassion Scale revised by Gong et al. (2014), which was adapted and localized from the original Self-Compassion Scale developed by (Neff 2003b). This scale consists of 12 items designed to assess three dimensions: self-kindness, common humanity, and mindfulness. A Likert 5-point scale was used, ranging from “strongly disagree” to “strongly agree,” with items 2, 4, 5, 8, and 11 being reverse-scored. The mean score of all items represented the overall level of self-compassion. The measurement outcomes demonstrated that the K-S non-parametric test reached a significant level (*p* < 0.001, *df* = 66), and the overall Cronbach's α coefficient was 0.743.

#### 2.3.4 Positive body image measurements

The multidimensional nature of the positive body image construct suggests that no single scale can currently provide a comprehensive assessment. However, existing research has demonstrated that body appreciation, as an operationalized construct, defined as affirming and accepting one's body, respecting its needs, and rejecting unrealistic appearance ideals promoted by the media as the sole standard of beauty (Tylka and Wood-Barcalow, 2015a), provides the most accurate and closest measurement of the core structure of positive body image (Swami et al., 2020). Nevertheless, the Body Appreciation Scale does not encompass all the characteristics of positive body image. Therefore, in scientific research, scholars often combine it with the Functionality Appreciation Scale, measuring from two perspectives: “appreciation of the body's unique features, health, and beauty” and “appreciation for what the body can do or is capable of doing (Alleva et al., 2017),” to achieve a reliable and effective evaluation of positive body image.

The study utilized the Chinese version of the Body Appreciation Scale-2 (BAS-2), originally revised by (Tylka and Wood-Barcalow 2015b) and later adapted by Swami et al. (2016). This scale consists of 10 items, measured using a Likert 5-point scale ranging from “strongly disagree” to “strongly agree,” with the overall mean score indicating the level of body appreciation. The measurement outcomes demonstrated that the K-S non-parametric test reached a significant level (*p* < 0.001, *df* = 45), and the overall Cronbach's α coefficient was 0.929.

Additionally, the study utilized the Chinese version of the Functionality Appreciation Scale (FAS), adapted by He et al. (2023), which was developed by Alleva et al. (2017). This scale consists of 7 items, measured using a Likert 5-point scale ranging from “strongly disagree” to “strongly agree,” with the overall mean score indicating the level of functionality appreciation. The measurement outcomes demonstrated that the K-S non-parametric test reached a significant level (*p* < 0.001, *df* = 21), and the overall Cronbach's α coefficient was 0.933.

### 2.4 Machine learning

This study utilized a Random Forest Regression model to predict positive body image among female university students. The model training process incorporated the following settings: Feature selection—to enhance the complexity of the machine learning model training, five independent variables were used as input features, including physical activity level, university student physical exercise, experience of embodiment, self-compassion and self-objectification. Number of trees—the model utilized 500 trees (*n* tree = 500) to improve the model's stability and accuracy. Feature selection at each split—at each tree split, one feature was randomly selected (*m* try = 1) to enhance the model's diversity and prevent overfitting. The model's performance was evaluated using Root Mean Square Error (RMSE) and the Coefficient of Determination (*R*^2^), where RMSE measures the prediction error and *R*^2^ assessed the model's explanatory power for the target variable.

This study utilized the XGBoost regression model to predict positive body image among female college students. The key parameters configured during the training process were as follows: Objective function—the model employed mean squared error (reg: squared error) as the loss function, which was appropriate for regression tasks. Number of trees—the model was configured with 100 iterations (*n* rounds = 100), where each iteration introduced a new tree to refine the model's predictions. Learning rate—this parameter regulated the contribution of each tree to the final model, thereby mitigating overfitting. For the data input, the model was trained using the same five features as the random forest: physical activity level, university student physical exercise, experience of embodiment, self-compassion, and self-objectification. In the model evaluation phase, the model's performance was evaluated using Root Mean Squared Error (RMSE) and the coefficient of determination (*R*^2^).

### 2.5 Statistical analyses

All core statistical analyses were conducted using SPSS version 29.0, with Excel utilized for data entry and preprocessing. Initially, the reliability and validity of the measurement instruments were assessed, followed by normality testing for the dataset. To address potential common method bias, Harman's single-factor test was performed using unrotated principal component analysis, with the threshold set at the first factor explaining less than 40% of the total variance. For baseline equivalence analysis, pre-test data were examined through normality assessment and Levene's test for homogeneity of variances. When both assumptions were satisfied, independent-samples t-tests were conducted to assess group equivalence on key variables. To evaluate the intervention effects, repeated-measures analysis of variance(ANOVA) was employed to test the main effects of time and group, as well as their interaction. When a significant interaction effect was detected, simple effects analyses were performed to examine between-group differences at each time point and within-group changes across time. To investigate associations among variables, Pearson product-moment correlation analyses were conducted using post-test data. Prior to mediation analysis, multicollinearity diagnostics were performed to ensure the absence of collinearity among independent variables. A chain mediation model (Model 6) was tested using Hayes' PROCESS macro for SPSS. Residual independence in the regression models was evaluated using the Durbin-Watson statistic. The significance of indirect effects was evaluated through bias-corrected bootstrap procedures with 5,000 resamples and 95% confidence intervals.

## 3 Results

### 3.1 Descriptive statistics

The pre-test data were subjected to normality and Levene's tests, which showed that the experience of embodiment, self-compassion, and positive body image scores of female college students followed a normal distribution and met the assumption of homogeneity of variance. Therefore, an independent samples *t*-test was conducted to assess the homogeneity of the experimental variables prior to the intervention. To further ensure that there were no significant differences between the basketball and comparison groups in terms of age, BMI and physical exercise level, an independent samples *t*-test was conducted on the demographic variables and physical exercise level of the two groups before the experiment. The results indicated that there were no significant statistical differences between the basketball and comparison groups in demographic variables and physical exercise level metrics, confirming that the two groups were comparable at baseline.

All variable measurements in this study were obtained through participants' self-reports, which may introduce common method bias. Therefore, Harman's single-factor analysis was used to test for this, conducting an unrotated exploratory factor analysis on all variables. The results showed that there were 14 factors with eigenvalues greater than 1, with the first factor explaining 28.17% of the variance, well below the critical threshold of 40%, indicating no significant common method bias issue in this study.

### 3.2 Difference test

Independent samples *t*-tests were conducted on the levels of the experience of embodiment, self-compassion, and positive body image for both the basketball and comparison groups in the pre-test, and post-test, as detailed in Table 2. The results indicated that there were no significant differences in the baseline levels of the three variables between the two groups before the experiment. However, after the experiment, the levels of all variables in the basketball group were higher than those in the control group.

**Table 2:** The assessment of participants' the experience of embodiment, self-compassion, and positive body image was conducted in pre-test and post-test.

**Variables**	**Time**	**Group**	**M ±SD**	***t***	***p***	**Cohen's *d***
Experience of embodiment	Pre-test	BG	84.67 ± 5.54	−0.31	0.76	0.09
		CG	85.10 ± 3.13			
	Post-test	BG	98.05 ± 4.75	2.850	0.007^**^	0.880
		CG	93.57 ± 5.41			
Self-compassion	Pre-test	BG	3.18 ± 0.28	0.053	0.96	0.04
		CG	3.17 ± 0.23			
	Post-test	BG	3.74 ± 0.24	3.486	0.001^**^	1.076
		CG	3.50 ± 0.20			
Positive body image	Pre-test	BG	3.57 ± 0.14	1.62	0.12	0.51
		CG	3.49 ± 0.17			
	Post-test	BG	4.11 ± 0.14	4.158	<0.001^**^	1.283
		CG	3.88 ± 0.21			

^*^BG, Basketball Group; CG, Comparison Group; M, mean; SD, standard deviation; ^*^p < 0.05; ^**^p < 0.01.

### 3.3 Effect of basketball intervention on the positive body image

This study utilized a two-factor, two-level repeated measures ANOVA design [2 (basketball group, comparison group) × 2 (pre-test, post-test)] to investigate the impact of group and time factors on various variable levels among female college students, as detailed in Table 3. The results indicated a statistically significant effect of the time factor (pre-test, post-test). Specifically, significant changes over time were observed in the levels of the experience of embodiment (*F* = _361.34_, *p* < 0.001, η^2^*p* = 0.90), self-compassion (*F* = _203.3_, *p* < 0.001, η^2^*p* = 0.84), and positive body image (*F* = _300.52_, *p* < 0.001, η^2^*p* = 0.88) in both the basketball and comparison groups. Additionally, the group factor (basketball group, comparison group) demonstrated statistical significance, revealing notable differences in the levels of the experience of embodiment (*F* = _4.63_, *p* < 0.05, η^2^*p* = 0.10), self-compassion (*F* = _2.22_, *p* < 0.05, η^2^*p* = 0.12), and positive body image (*F* = _7.82_, *p* < 0.01, η^2^*p* = 0.16) between the two groups. Furthermore, the interaction between time and group was significant, indicating that the extent of change over time in the levels of the experience of embodiment (*F* = _45.91_, *p* < 0.001, η^2^*p* = 0.53), self-compassion (*F* = _13.81_, *p* < 0.001, η^2^*p* = 0.26), and positive body image (*F* = _3.92_, *p* < 0.05, η^2^*p* = 0.09) differed significantly between the basketball and comparison groups.

**Table 3:** Analysis of variance on the effect of basketball intervention on various variable levels in female college students.

**Variables**	**Sources of variation**	**Type III sums of squares**	***df***	**Mean square**	***F***	***p***	**η^2^*p***
Experience of embodiment	Time	1943.058	1	1943.05	361.34	<0.001	0.90
	Error	215.10	40	5.38			
	Group	189.00	1	189.00	4.63	<0.05	0.10
	Error	1633.95	40	40.85			
	Time × Group	246.86	1	246.86	45.91	<0.001	0.53
Self-compassion	Time	4.24	1	4.24	203.3	<0.001	0.84
	Error	0.84	40	0.02			
	Group	0.31	1	0.31	2.22	<0.05	0.12
	Error	3.72	40	0.10			
	Time × Group	0.29	1	0.29	13.81	<0.01	0.26
Positive body image	Time	4.96	1	4.96	300.52	<0.001	0.88
	Error	0.66	40	0.02			
	Group	0.38	1	0.38	7.82	<0.01	0.16
	Error	1.95	40	0.05			
	Time × Group	0.07	1	0.07	3.92	<0.05	0.09

When the interaction effect was found to be significant, further simple effect analysis was conducted, as detailed in Table 4. Specifically, during the pre-test, no significant statistical differences were found in the experience of embodiment, self-compassion, and positive body image among female college students in different groups, suggesting that the levels of these variables were comparable between the basketball and comparison groups at baseline. In the post-test, however, significant statistical differences were observed in the experience of embodiment, self-compassion, and positive body image among female college students in different groups, indicating that there were significant differences in the levels of these variables between the basketball and comparison groups. Within the basketball group, significant statistical differences were observed in the experience of embodiment, self-compassion, and positive body image across different time factors, demonstrating that the levels of these variables changed significantly from the pre-test to the post-test. Similarly, within the comparison group, significant statistical differences were observed in the experience of embodiment, self-compassion, and positive body image across different time factors, indicating that the levels of these variables also changed significantly from the pre-test to the post-test, though to a lesser extent compared to the basketball group.

**Table 4:** The simple effect analysis of the interaction between time and group on the variable levels of female college students.

**Variables**	**Simple main effect**	**Mean deviation**	**Standard deviation**	***t***	***p***
Experience of embodiment	Pre-test	−0.429	1.387	−0.309	0.76
	Post-test	6.429	1.574	4.084	<0.001
	Basketball group	−13.048	0.716	−18.232	<0.001
	Comparison group	−6.190	0.716	−8.650	<0.001
Self-compassion	Pre-test	0.004	0.079	0.055	0.957
	Post-test	0.239	0.068	3.486	0.001
	Basketball group	−0.567	0.045	−12.710	<0.001
	Comparison group	−0.332	0.045	−7.455	<0.001
Positive body image	Pre-test	0.079	0.049	1.598	0.12
	Post-test	0.190	0.061	3.101	<0.01
	Basketball group	−0.541	0.040	−13.657	<0.001
	Comparison group	−0.430	0.040	−10.859	<0.001

**Figure 1:**
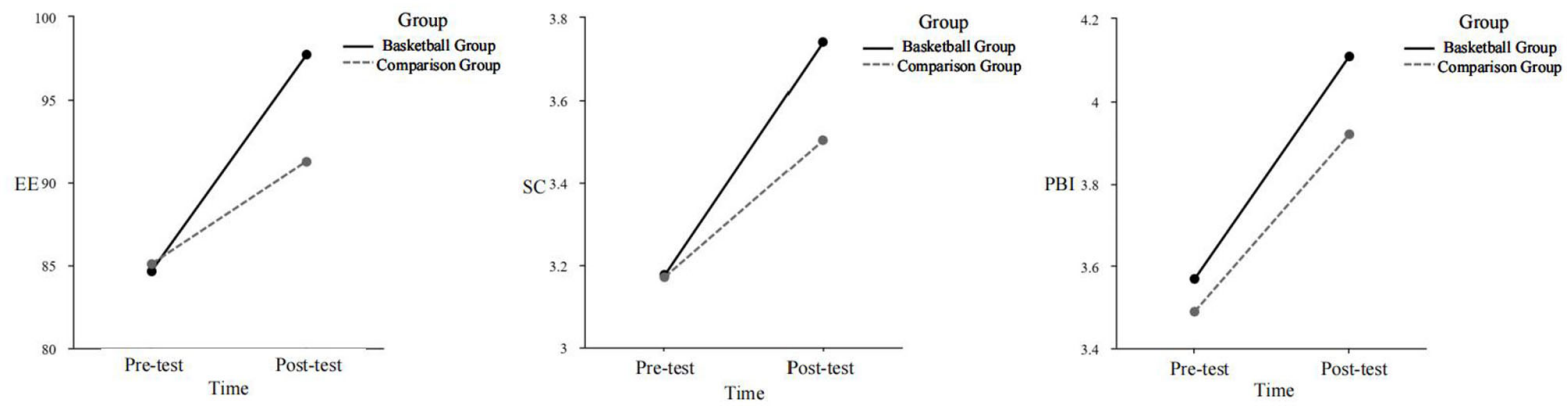
Changes in the levels of various variables across all groups before and after the experiment. EE, Experience of Embodiment; SC, Self-compassion; PBI, Positive Body Image.

Multiple comparisons and simple slope analyses, as shown in Figure 1, revealed that after a 10-week basketball intervention, the post-test levels of various variables in female college students in the basketball group were higher than their pre-test levels and significantly exceeded those of the comparison group, highlighting the effectiveness of the basketball intervention in improving these psychological and physical well-being measures.

### 3.4 Mediating effect test

The post-test data from the study were analyzed using Pearson's bivariate two-tailed correlation tests to examine the relationships between basketball intervention, experience of embodiment, self-compassion, and positive body image, as showed in Table 5. The results revealed significant positive correlations (*p* < 0.01) among basketball intervention, experience of embodiment, self-compassion, and positive body image.

**Table 5:** Correlation analysis (post-test).

**Variables**	**1**	**2**	**3**	**4**
1. Basketball intervention	1			
2. Experience of embodiment	0.41^**^	1		
3. Self-compassion	0.48^**^	0.49^**^	1	
4. Positive body image	0.55^**^	0.36^**^	0.73^**^	1

^*^p < 0.05; ^**^p < 0.01.

The results of the correlation analysis indicated significant associations among the variables, which could potentially lead to multicollinearity issues, thereby affecting the accuracy of regression analysis. To mitigate this, the predictor variables in each equation were standardized, and multicollinearity tests were performed. The results indicated that the tolerance of all predictor variables ranged from 0.55 to 0.93 (greater than 0.1), and the Variance Inflation Factor(VIF) ranged from 1.08 to 1.81 (less than 5). These findings confirm that the data in this study do not exhibit multicollinearity issues, satisfying the necessary conditions for testing mediation and chain mediation effects.

This study utilized post-test data and employed the SPSS plugin Process developed by (Hayes 2017) to construct and test the chain mediation model, selecting Model 6 for analysis. The group factor was set as a binary independent variable (comparison group coded as “0”, basketball group coded as “1”), with positive body image as the dependent variable, experience of embodiment and self-compassion as chain mediators, and BMI as a control variable. Multiple linear regression analyses were conducted with positive body image as the dependent variable and Basketball Intervention, Experience of Embodiment, and Self-Compassion as independent variables, respectively. The D.W values ranged from 1.61 to 1.77, falling within the acceptable range of 1.5–2.5, indicating no first-order residual auto correlation. The regression analysis results, detailed in Table 6, demonstrated that basketball intervention significantly and positively predicts positive body image (β = 0.48, *t* = 3.59, *p* < 0.01). Additionally, basketball intervention significantly and positively predicts the experience of embodiment (β = 0.39, *t* = 2.55, *p* < 0.05) and self-compassion (β = 0.30, *t* = 2.05, *p* < 0.05). Further analysis revealed that the experience of embodiment does not significantly predict positive body image, but it indirectly enhances positive body image by eliciting self-compassion, forming a complete chain mediation pathway (β = 0.59, *t* = 4.56, *p* < 0.01). This indicates that the effect of the experience of embodiment depends on the psychological mechanism of self-compassion, as enhanced bodily perception may ultimately improve positive body image through increased self-acceptance and self-kindness.

**Table 6:** Regression analysis between variables (post-test/standardized).

**Variables**	**Positive body image (1)**		**Experience of embodiment**		**Self-compassion**		**Positive body image (2)**	
	β	***t***	β	***t***	β	***t***	β	***t***
BMI	0.27	2.04^*^	0.09	0.61	0.19	1.39	0.15	1.32
Basketball intervention	0.48	3.59^**^	0.39	2.55^*^	0.30	2.05^*^	0.25	2.02^*^
Experience of embodiment					0.33	2.28^*^	0.05	0.42
Self-compassion							0.59	4.56^**^
*R^2^*	0.31		0.18		0.37		0.61	
*ΔR^2^*	0.27		0.13		0.34		0.56	
*DW*	1.77		1.61		1.906		1.97	
*F*	8.57^**^		4.18^*^		11.39^**^		14.25^**^	

^*^p < 0.05; ^**^p < 0.01.

**Figure 2:**
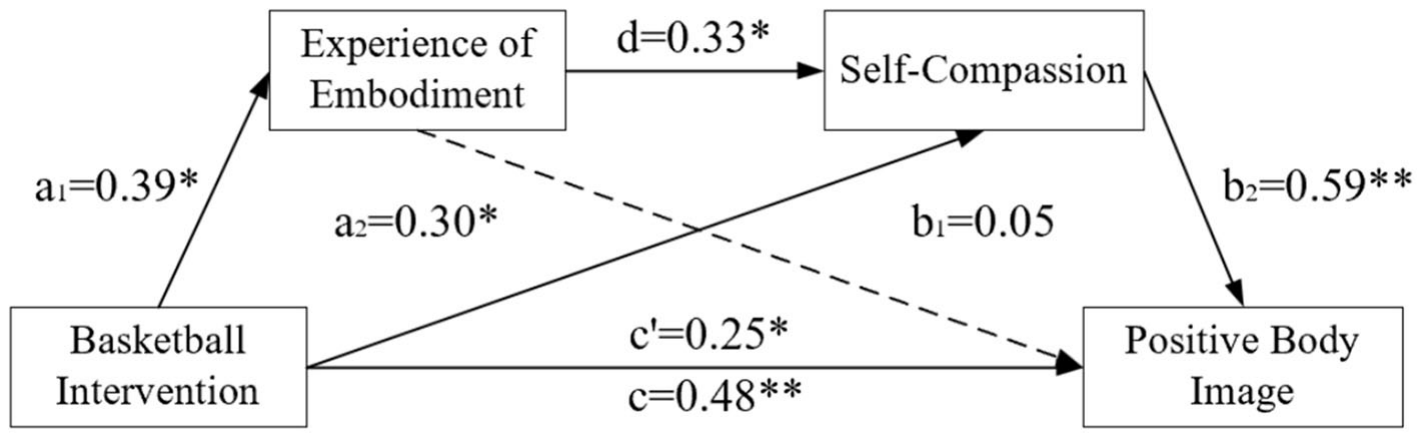
The chain mediation model. *p < 0.05; **p < 0.01. *a*_1_ predicts the regression coefficient of Basketball Intervention for Experience of Embodiment; *a*_2_ and d jointly predict the partial regression coefficient of Basketball Intervention and Experience of Embodiment; c', *b*_1_ and *b*_2_ jointly predict the partial regression coefficient of Positive Body Image for Basketball Intervention, Experience of Embodiment and Self-Compassion; *c* indicates the regression coefficient of Basketball Intervention to Positive Body Image without mediating variables.

According to the mediation effect testing procedure proposed by Wen et al. (2022), the first step examined the regression coefficient *c'* of basketball exercise intervention on positive body image (*c'* = 0.25, *t* = 2.02, *p* < 0.05), establishing the mediation effect of basketball exercise intervention on positive body image. The second step sequentially tested the coefficient *a*_1_ = 0.39, *a*_2_ = 0.30; the coefficient *b*_1_ = 0.05, *b*_2_ = 0.59, where coefficient *b*_1_was not significant. The resulting model and the path relationships between variables are illustrated in Figure 2.

In the third step, the mediation effect was tested using the bias-corrected non-parametric percentile Bootstrap method, with 5,000 resampling iterations, and the 95% Confidence Interval(CI) was calculated, as detailed in Table 7. The mediation effect value of the experience of embodiment was 0.013, with a 95% *CI* of [−0.005, 0.031]. Since the interval includes 0, it indicated that the independent mediation effect was not significant. The mediation effect value of self-compassion was 0.160, with a 95% *CI* of [0.104, 0.215]. The chain mediation effect value of the two mediators was 0.057, with a 95% *CI* of [0.034, 0.079]. Since neither interval includes 0, it indicated that the mediation effects of self-compassion and the chain mediation were significant. Further decomposing the effect sizes of each variable on positive body image, the direct effect of basketball intervention on positive body image was 0.246, while the total indirect effect value of 0.230 was the sum of the mediation effects of the three mediation paths. The sum of the direct effect and the total indirect effect gave the total effect of 0.476. The relative effect proportions were calculated by dividing each mediation effect value by the total effect. The effect proportions of the three mediation paths in this study were 2.73%, 33.61%, and 11.98%.

**Table 7:** Test of the mediating effect.

**Path**	**Effect**	**Boot SE**	**Boot95%CI**		**Relative mediating effect**
			**UB**	**LB**	
BI → EE → PBI	0.013	0.001	−0.005	0.031	2.73%
5BI → SC → PBI	0.160	0.028	0.104	0.215	33.61%
BI → EE → SC → PBI	0.057	0.012	0.034	0.079	11.98%
Total indirect effect	0.230	0.059	0.115	0.345	48.32%
Direct effect	0.246	0.064	0.121	0.370	51.68%
Total effect	0.476	0.076	0.328	0.624	100.00%

BI, Basketball Intervention; EE, Experience of Embodiment; SC, Self-compassion; PBI, Positive Body Image; LB, Lower Boundl; UB, Upper Bound.

### 3.5 Machine learning test

The Random Forest Regression model was employed to predict positive body image, with independent variables including physical activity level, university student physical exercise, the experience of embodiment, self-compassion and self-objectification. The training results of the model was as follows. Model Performance: The model's Mean Squared Error (MSE) was 0.0052, indicating a small prediction error and high predictive accuracy. The model's *R*^2^ value was 0.764, meaning that the model can explain approximately 76.4% of the variance in positive body image, demonstrating a strong fit for the dependent variable. In the interpretation of variable importance, self-compassion ranks first, followed closely by physical activity level, as shown in Table 8.

**Table 8:** The results of random forest model.

**Variables**	**IncNodePurity**
Self-compassion	0.224
Physical activity level	0.161
Experience of embodiment	0.134
University student physical exercise	0.125
Self-objectification	0.124

Subsequently, the robustness of the model's results was enhanced by using the XG Boost algorithm. The results showed an RMSE of 0.1488, which represented the root mean square error on the test data, indicating the difference between the model's predicted values and the actual values. This RMSE value suggested that, while the model performed excellently on the training data, it also maintained a good performance on the test data, demonstrating minimal overfitting. The *R*^2^ value of 0.8732 measured the model's ability to explain the variation in the target variable (positive body image). This *R*^2^ value indicated that the model explained approximately 87.32% of the variance in the target variable. While the *R*^2^ value was relatively high, it was important to note that the model did not perfectly fit the training data, but rather demonstrated a good balance between accuracy and generalization. The contribution of each independent variable to the prediction results was analyzed. Feature importance was evaluated through the model's Gain, Cover and Frequency metrics, as shown in Table 9. According to the XG Boost output, the ranking of feature importance in predicting positive body image was as follows: self-compassion contributed the most to the model's prediction, with a Gain value of 0.332, indicating that it contributed the most explanatory power in all tree splits. Physical activity level and college student physical exercise also had significant impacts on the prediction, ranking second and third with Gains of 0.263 and 0.186, respectively. The contributions of self-objectification and the experience of embodiment were smaller, at 0.150 and 0.069, respectively.

**Table 9:** The results of XGboost model.

**Features**	**Gain**	**Cover**	**Frequency**
Self-compassion	0.3321	0.14997	0.18398
Physical activity level	0.2627	0.1547	0.2511
University student physical exercise	0.1860	0.2966	0.2338
Self-objectification	0.1504	0.2470	0.1732
Experience of embodiment	0.0687	0.1518	0.1580

## 4 Discussion

This study explored the effects of a 10-week basketball intervention on female college students' experience of embodiment, self-compassion, and positive body image, and analyzed the chain mediating roles of the experience of embodiment and self-compassion in this process. The results demonstrated that the basketball intervention significantly enhanced the experience of embodiment, self-compassion, and positive body image among female college students, with the basketball group showing significantly greater improvements compared to the comparison group. Further investigation into the mechanism of the basketball intervention's impact on positive body image revealed that the independent mediating effect of the experience of embodiment was not significant, while the mediating effect of self-compassion was significant. Additionally, the chain effect of both factors was significant, indicating that the basketball intervention promotes the development of positive body image through the sequential pathways of experience of embodiment and self-compassion. The study also employed Random Forest and XG Boost models to predict positive body image. The results showed that self-compassion contributed the most to the prediction, followed by physical activity level and student exercise involvement.

At this stage, numerous scholars have empirically confirmed the positive impact of physical exercise on body image through qualitative, quantitative, and mixed-method research. However, due to variations in intervention programs in terms of content, frequency, and duration, a unified research conclusion has yet to be established (Halliwell et al., 2018). It is noteworthy that the basketball intervention in this study demonstrated a significant positive effect on positive body image (β = 0.48), with the effect size classified as moderate to strong. Compared with previous studies, a 12-week CrossFit intervention yielded effect sizes (β = 0.22–0.36) across different dimensions of positive body image (Swami, 2019), while an 8-week Functional Training intervention showed effects (β = 0.43) on body appreciation (Jankauskiene et al., 2024). A 4-week online yoga plus gratitude journaling intervention produced effects (β = 0.54) on body appreciation (Jennifer et al., 2022). The effect size observed in the current study therefore ranks moderately high within the existing literature. This result aligns with prior findings that physical exercise promotes positive body image (Burgon and Waller, 2024). It also supports the Embodied Cognition Theory, which posits that enhancing bodily proprioception through exercise helps regulate individuals' cognitive and emotional experiences of their bodies (Zhang and Ji, 2023). From the characteristics of basketball, it effectively encourages participants to focus on body strength, coordination, and athletic performance, which reduces excessive attention to body appearance. Nevertheless, there remains a lack of comparative research on the intervention effects of different types of sports on positive body image, although the effectiveness of exercise interventions has been confirmed in activities such as yoga, dance, and fitness training (Wong et al., 2021a,b). Notably, existing studies have found that athletes in aesthetic or lean sports report higher levels of body dissatisfaction and negative body image (Burgon et al., 2023). Therefore, compared to sports like gymnastics, dance, and skating that emphasize body shape control, the competitive nature of basketball may more effectively challenge women's stereotypical perceptions of a “fragile body,” thereby fostering the development of positive body image.

Existing research has confirmed a significant positive correlation between physical exercise and experience of embodiment. Women who engage in higher levels of physical activity tend to demonstrate stronger experience of embodiment during exercise (Paulson and Greenleaf, 2022). However, there is still limited experimental evidence verifying the causal impact of physical exercise on the experience of embodiment. In the experimental design of this study, grounded in Cognitive Learning Theory, basketball skill objectives, and psychological education goals were structured across levels of perception, comprehension, memorization, and innovation. Diverse teaching methods were employed to guide participants in enhancing their connection with their bodies and in fostering subjective agency through the learning and control of movement. Within the exercise environment, the implementation of intervention programs and teacher guidance enabled participants to focus on and appreciate their bodily functions rather than external features, thereby effectively improving their level of experience of embodiment (Daniels et al., 2020). However, the effect of experience of embodiment as a single mediating variable on positive body image did not reach significance. This may be due to two reasons. First, it is related to the six dimensions included in the Embodied Experience Scale not being fully applicable to the exercise context. For example, the dimension “Experience and Expression of Sexual Desire (EESD)” has limited applicability in exercise settings, which may have affected the assessment of the intervention effect. Second, as an immediate bodily perception in exercise contexts, embodied experience does not always present a positive state. When individuals fail to complete movements, different psychological states, such as self-compassion and self-criticism, exert different effects on sport participation (Walter and Heinen, 2019) and body image. Therefore, regulation through positive psychological resources is required to transform this experience into positive body image, which further highlights the important influence of self-compassion as a psychological resource in body image interventions. Accordingly, future research could refine the item statements of the scale to enhance its applicability in exercise contexts. At the same time, exploring extended intervention durations could test whether experience of embodiment develops a direct effect as self-compassion resources accumulate.

Physical exercise plays a significant role in reducing vulnerability and psychological distress (Vella et al., 2019) and is therefore widely regarded as an effective means of enhancing self-compassion. Existing research has shown that physical exercise can significantly improve individual self-compassion levels, but current studies primarily focus on activities such as Yoga and Tai Chi, with relatively less attention given to other sports (Wong et al., 2021b). Among self-compassion intervention methods, the Mindful Self-Compassion (MSC) program developed by scholars such as Neff has gained widespread recognition for its comprehensive structure and notable effectiveness (Neff and Germer, 2013). This study integrates MSC unit themes with basketball teaching objectives to construct a multidimensional self-perception and emotional support system for participants. The results indicate that physical exercise significantly promotes self-compassion, which aligns with the intervention effects of combining the MSC method with aerobic exercise (Bluth and Eisenlohr-Moul, 2017; Dai and Liu, 2022). Notably, non-verbal communication in basketball, such as high-fives for encouragement, can more effectively reinforce the behavioral expression of self-compassion compared to mind-body connected sports (Sun and Chen, 2021). This embodied experience has a longer-lasting impact than cognitive training alone. When self-compassion serves as a single mediating variable, it significantly predicts the impact on positive body image. This may be due to the reconstruction of social comparison tendencies among female college students during basketball participation. Uniform team attire diminishes focus on physical appearance differences, while movement practice and application shift body evaluation from an aesthetic dimension to a functional one (Fougner et al., 2019). These cognitive transformation mechanisms provide a mediating pathway for the development of positive body image through self-compassion.

The findings of this study indicate that the experience of embodiment and self-compassion play a significant chain-mediated role between basketball intervention and positive body image. Basketball, through dynamic physical engagement such as running, jumping, and collaboration, shifts attention from body appearance to body functionality, such as strength, coordination, and endurance, thereby fostering embodied cognition. Simultaneously, the endorphins and dopamine released during exercise significantly enhance mood, and the sense of achievement from completing technical movements reinforces positive body perception. The experiences and emotions gained through the interaction of the body with the environment during exercise also provide individuals with opportunities to re-examine and understand their own bodies. This enhances the experience of embodiment and reduces self-objectification tendencies (Tiggemann et al., 2011). Additionally, the flexibility and functionality demonstrated during exercise offer diverse dimensions for self-evaluation, helping individuals transcend singular aesthetic standards and form a more inclusive and positive body image. However, the experience of embodiment in exercise is not always positive. Negative situations such as mistakes or failures provide opportunities for the development of self-compassion, encouraging individuals to treat their shortcomings with tolerance and acceptance (Mosewich et al., 2019). This self-compassion-based mindset helps individuals maintain a positive psychological state in the face of setbacks and further consolidates and develops a positive body image. In summary, basketball intervention enhances individuals' positive perception of their bodies by reinforcing the experience of embodiment and reducing excessive focus on appearance. At the same time, the cultivation of self-compassion enables individuals to face challenges and setbacks in exercise with a more inclusive and accepting attitude, thereby further promoting the formation and development of a positive body image. This dual-dimensional mechanism provides a more comprehensive theoretical explanation for understanding the improvement of mental health through exercise intervention.

Although this study has revealed the impact of basketball on the positive body image of female college students and its underlying mechanisms, certain limitations remain. First, the research sample was confined to female students from two universities in China. This limited geographical coverage and the exclusion of male participants restrict the generalizability of the conclusions and prevent the analysis of gender differences. Second, the 10-week intervention period was insufficient to observe long-term effects. The exclusive focus on basketball as the intervention activity, while the control group received a mixed-activity physical education program, made it difficult to determine whether basketball's effects were due to its team-based characteristics, exercise intensity, or other factors. The absence of comparisons with other individual sports such as swimming or aerobics may obscure the unique effects of different physical activities on positive body image. Third, although the basketball intervention significantly improved all target variables, the time × group interaction effect on positive body image showed a small effect size. Future studies with larger samples will improve the power to detect such complex pathways. Additionally, the inadequacy of some scales in the context of sports may weaken the measurement accuracy of core variables such as the experience of embodiment. This study primarily focused on the chain mediation effects of the experience of embodiment and self-compassion, without incorporating potential influencing factors like social support and family background, which limits the comprehensiveness of the mechanism explanation. Future research should expand the sample to include multiple regions and types of universities, conduct cross-gender comparisons to reveal moderating effects, and extend the intervention period to over 6 months, incorporating follow-up studies to examine the sustainability of effects. It should also introduce diverse sports for horizontal comparison to clarify the differential impacts of various physical activities, optimize measurement tools to better suit their application in sports contexts, and integrate multimodal data such as physiological indicators and behavioral observations to enhance assessment validity. Furthermore, a dynamic mediation model should be constructed by incorporating variables such as social support to refine the mechanism explanation framework. Ultimately, based on empirical findings, gender-specific and sport-specific intervention programs should be designed to provide theoretical and practical foundations for sports in promoting mental health.

## 5 Conclusion

This study validated the effectiveness of a 10-week basketball intervention program in improving positive body image among female university students, while also clarifying the underlying mechanisms. The basketball group showed significantly greater improvements in the experience embodiment, self-compassion, and positive body image compared to the mixed-activity standard physical education program. This confirmed that participation in basketball promoted the development of a positive body image by reinforcing the chain-mediated pathways of the experience of embodiment and self-compassion. The core mechanisms of the intervention included team collaboration and physical coordination training, which helped reduce appearance-focused preoccupation, along with positive attribution strategies that enhanced psychological resilience. As a moderate-to-high-intensity team sport, basketball proved effective in improving mental health outcomes among female college students. The identified dual “body-mind” pathway mechanism provided empirical support for strategies promoting campus sports. These findings indicated that structured sports interventions should be incorporated into university mental health education systems to offer holistic support for students' psychological well-being through physical activity participation.

## Data Availability

The raw data supporting the conclusions of this article will be made available by the authors, without undue reservation.
